# Effect of Postural Stabilization Exercises in Combination with Cervical Stabilization Exercises on Craniovertebral Angle, Pain, Disability, and Quality of Life in Patients with Chronic Neck Pain: A Randomized Controlled Trial

**DOI:** 10.3390/healthcare13121388

**Published:** 2025-06-11

**Authors:** Gölgem Mehmetoğlu, İnci Yüksel

**Affiliations:** 1 Department of Physiotherapy and Rehabilitation, Faculty of Health Sciences, European University of Lefke, Northern Cyprus TR-10 Mersin, Lefke 99728, Turkey; 2 Department of Physiotherapy and Rehabilitation, Faculty of Health Sciences, Eastern Mediterranean University, Northern Cyprus, Mersin 10, Gazimagusa 99628, Turkey; inci.yuksel@emu.edu.tr

**Keywords:** chronic neck pain, craniovertebral angle, lumbopelvic muscle exercises, scapular stabilization, postural stabilization

## Abstract

**Objective:** The aim of the study was to evaluate the effect of postural stabilization exercises, in addition to cervical stabilization (CS), on the craniovertebral angle (CVA), pain, neck disability index (NDI), and quality of life in people with chronic neck pain. **Methods:** This study was performed on 60 women with chronic neck pain, aged 20–60 years, who were randomly divided into two groups. Individuals in the first group underwent CS exercises, in addition to scapular and lumbopelvic stabilization (SLPS) exercises, three times a week for 6 weeks. The individuals in the second group underwent only CS exercises for the same period. Moreover, stretching exercises and a 20 min hot pack were applied to the muscles around the neck of all patients. The CVA was measured using photogrammetry. The visual analog scale (VAS) was used to assess pain. The disability level was measured using the NDI. The Turkish version of the 36-Item Short-Form Health Survey (SF-36) was used to assess quality of life. The assessments were conducted before treatment, after treatment, and at the 2-month follow-up. To assess changes over time and between groups, a two-way repeated measures analysis of variance (ANOVA) was conducted. **Results:** Post-treatment measurements revealed significant differences favoring the SLPS group. The VAS and NDI scores were markedly lower in the SLPS group than in the CS group, both post-treatment (*p* < 0.001) and at follow-up (*p* < 0.001). The CVA was significantly greater in the SLPS group at both the post-treatment (*p* < 0.001) and follow-up (*p* < 0.001) assessments. However, in all sub-parameters except the SF-36 general health subscale, the SLPS group reported higher scores than the CS group post-treatment and at follow-up. Effect sizes for between-group comparisons ranged from moderate to very large (Cohen’s d = 0.65 to 2.31), and partial eta-squared (η^2^) values indicated moderate to large effect magnitudes (η^2^ = 0.09 to 0.48), supporting the clinical relevance of the findings. **Conclusion:** In individuals with neck pain, including SLPS exercises in the treatment program, rather than just exercises for the cervical region, provides more positive results in terms of reducing disability and pain and increasing functionality. Clinical Trial Registration Number: NCT06578481.

## 1. Introduction

Neck pain is a common musculoskeletal condition [1] characterized by tension, fatigue, or pain in the neck area, which may radiate to the shoulders, arms, and head. It often results in considerable disability and poses a significant socioeconomic burden [2]. Neck pain is more common in women than in men, which causes women to take time off from work [1]. It mostly affects people with ages of 18–29 and 40–49 [1]. Women have smaller neck muscles and weaker cervical structures; therefore, they are more prone to strain and injury. This can lead to ligament laxity, joint instability, and pain [1,3].

Protracted shoulders and forward head displacement are frequent in people with chronic neck pain compared with those without symptoms [4]. Alterations in the length–tension correlation of the muscles connecting the scapula, head, cervical spine, and chest lead to neck pain. Furthermore, changes in the neuromuscular control of muscles, including the trapezius, levator scapulae, and rhomboid minor, which are directly connected to the cervical spine, induce compressive and shearing forces in the neck region that cause pain. The major risk factors for chronic neck pain are changes in muscle activation patterns and scapular posture [4].

Chronic neck pain leads to various muscle activation patterns and movement mechanics, which may result in reduced muscle strength [4,5]. Also, compared with healthy controls, people with neck pain experience pain sensitivity and changes in the activity of the axioscapular muscle [6]. Neck stabilization exercises help in managing and preventing spinal dysfunction by training local muscles and reducing the over activity of superficial muscles [7].

Maintaining the mechanical stability of the spine is vital in managing neck and low back pain issues [8]. Previous findings indicated that, irrespective of the level at which the neck pain is experienced, spinal pain impacts trunk muscle control, resulting in changes in trunk muscle function associated with neck pain [9]. Furthermore, the performance of lumbar stabilization is abnormal in people with neck pain [10].

Columna vertebralis is a whole anatomical structure, so pain in one region may have a negative impact on other regions [11]. There is a significant positive correlation in degenerative changes between cervical and lumbar vertebrae. Patients with chronic neck pain were found to have abnormal performance of the lumbopelvic stabilizer muscles in the abdominal drawing-in maneuver despite having no complaints of low back pain [11,12].

Previous studies have investigated the effectiveness of cervical stabilization (CS), scapular stabilization, and combined cervical and scapular stabilization exercises in people with neck pain [2,4,13,14].

Neck stabilization exercises have been found to be effective when applied to individuals with chronic neck pain in reducing pain and disability [2]. In the literature, scapular stabilization exercises were found to be beneficial in reducing pain and dysfunction in individuals with chronic neck pain [13]. In a study in which scapular stabilization and neck stabilization exercises were applied together, it was found that they reduced pain and disability and improved forward head posture [4]. In another study, neck stabilization exercises and neck and scapular stabilization exercises were compared, and their effects on pain and disability were examined. It was found that pain and disability decreased in both groups, but there was no difference between the groups [14].

There are few studies reporting the results of the treatment of lumbopelvic stabilizer muscles in individuals with neck pain [8,10]. In one study, elderly individuals with chronic neck pain were treated with neck, core stabilization, and combined neck and core stabilization exercises. Pain reduction was found in all treatment groups [15]. In another study, lumbopelvic stabilization exercises and deep cervical flexion training were compared. It was found that lumbopelvic training was more useful for reducing pain and correcting posture [8].

People with chronic or recurrent neck pain may be unable to maintain head/neck position and postural stability. Hence, scapular and lumbopelvic stabilization (SLPS) exercises should be included in treatment programs in addition to exercises that help neck muscles to increase postural support. However, whether adding SLPS exercises to the CS program can change the treatment outcomes of people with neck pain remains unexplored. Therefore, this study was conducted to assess how postural stabilization exercises influence the craniovertebral angle, pain, neck disability index (NDI), and quality of life in individuals suffering from chronic neck pain.

## 2. Methods

### 2.1. Study Design

This was a randomized, comparative, and single-center study conducted in accordance with the principles of the Declaration of Helsinki. It was approved by the Eastern Mediterranean University Scientific Research and Health Ethics Committee (ETK00-2022-0210, 28 June 2022) and conducted in a private Physical Therapy and Rehabilitation Center in Güzelyurt between July and December 2022. The study was unblinded due to the nature of the intervention, as both the participants and the physiotherapist had to know the exercise protocols. For standardization, all the evaluations and interventions were carried out by the same physiotherapist, who had 7 years of experience in the field.

The study was registered on ClinicalTrials.gov with the identifier NCT06578481.

### 2.2. Participants

This study included people diagnosed with chronic neck pain by orthopedics and traumatology specialists. The sample size required for the study was determined using G*Power software (version 3.1.9.2). The effect size was found to be high (*d* = 2.27) based on the findings of a study by Kang-Seong Lee (2020) titled “Effect of a Five-week Scapular Correction Exercise in People with Chronic Mechanical Neck Pain” [16]. The sample size calculated through power analysis with an effect size of *f* = 1 and a significance level of α = 0.05 for 95% (1 − β = 0.95) power revealed that each group required 25 people. Considering that some participants might not follow the treatment protocols for various reasons, 30 people were included in each group.

#### Inclusion and Exclusion Criteria

As mentioned in the Introduction Section, neck pain is more common in women with ages of 18–29 and 40–49 [1]. Therefore, this study included women aged 20–60 years with moderate pain intensity [3 cm and above according to the visual analog scale (VAS)] and neck pain lasting more than 3 months.

The exclusion criteria were as follows: participants who had undergone any shoulder or neck surgery; those who complained of fibromyalgia; those who had a neurological disease, cervical radiculopathy, or serious systemic disease interfering with treatment; and those who were pregnant.

### 2.3. Randomization

**Figure 1:**
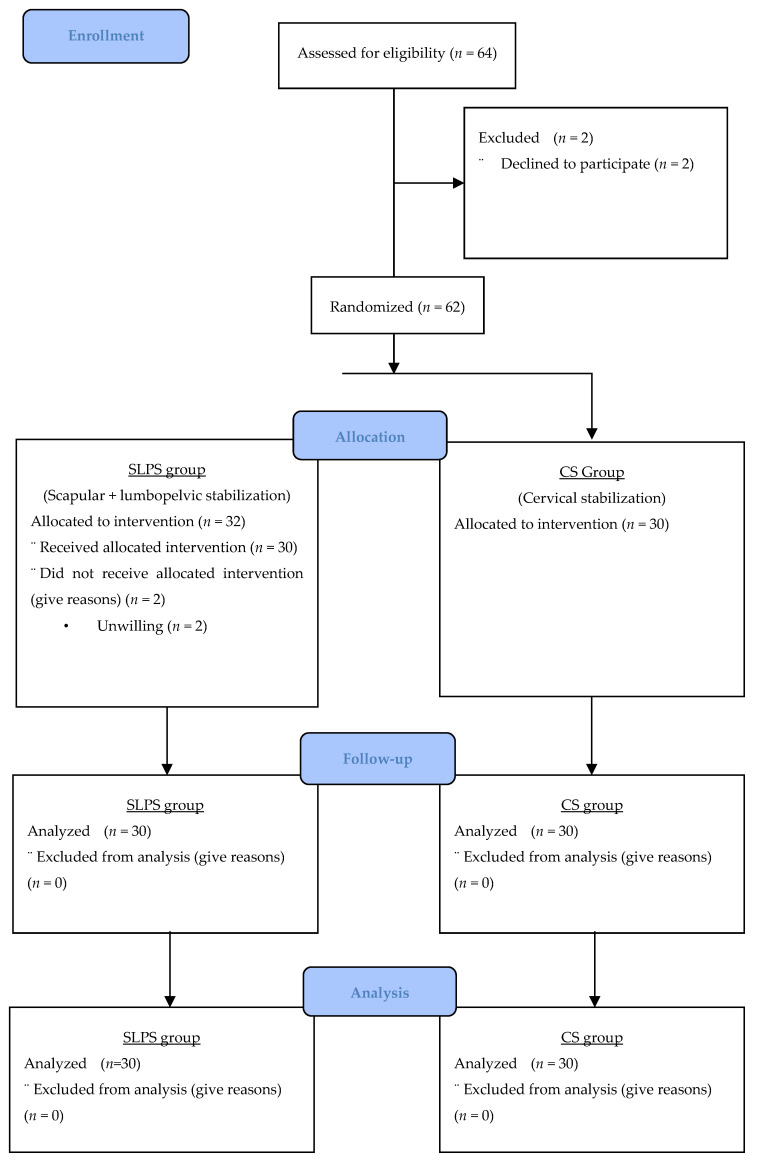
CONSORT flowchart of participant recruitment and retention.

Participants were randomly assigned by the principle investigator, who also performed the assessment and treatment, to one of two groups using the coin flip method: the SLPS group (*n* = 30) and the CS group (*n* = 30). The participant flowchart is shown in Figure 1 [17]. Participants were informed about the study before enrollment and signed written informed consent forms. Finally, 64 participants meeting the inclusion criteria were included in the study.

### 2.4. Interventions

The study included 64 people with chronic neck pain, 2 of whom dropped out of the study after the evaluation, as they did not want to participate. The 62 people included in the study were randomly divided into two groups. The first group (SLPS group, *n* = 32) received SLPS exercises in addition to CS exercises. The second group (CS group, *n* = 30) received only CS exercises. After the participants had started to receive treatment, two people from the SLPS group stopped participating in the study. The study was completed with 60 participants in total. The demographic characteristics of the participants are listed in Table 1. Participants received retraining under the supervision of a physiotherapist three times a week for 6 weeks. The evaluation was repeated three times as pre-treatment, post-treatment, and a follow-up evaluation 2 months after the end of treatment. All individuals received a 20 min hot pack application at the beginning of the treatment session.

#### 2.4.1. Cervical Stabilization Exercises

Deep cervical flexor muscle exercises: These exercises were performed using a stabilizer with an air-inflated pressure sensor (Stabilizer Pressure Biofeedback-ChattanooGA Stabilizer). The participants were laid on their backs in the supine position (without a pillow) to ensure the neck was in a neutral position. Each participant was treated with slow, controlled flexion of the head and upper cervical region, with the chin closely pressed to the chest (“yes” movement) to achieve craniocervical flexion and mild axial extension. The stabilizer was placed under the occiput and inflated to a pressure of 20 mm Hg. Gradual training was provided to increase the pressure to between 20 and 30 mm Hg by 2 mm Hg. This training was performed 10 times by holding for 10 s at each pressure value [18,19].

Chin tuck exercise: The participants were asked to stand upright without tilting the head forward, backward, or to the side and to pull the jaw back as if trying to remove food (15 repetitions).

Shoulder shrug exercise: The participants were asked to pull their shoulders toward their ears and release them while standing (15 repetitions). The exercise progressed by adding weight with dumbbells (three sets of 10 repetitions).

The participants were asked to perform eccentric flexion of the lower cervical spine in a prone position on their elbows while maintaining a neutral craniocervical spine. They were then asked to return to the starting position by slowly extending the lower cervical vertebrae.

#### 2.4.2. Scapular Stabilization Exercises

The participants were positioned kneeling with their knees flexed at 90° with a Pilates ball placed between the chest and the abdomen. From the side, the earlobe, scapula, acromion, and pelvis were aligned in a straight line. The participants were asked to perform the first three exercises below in this position. Two sets of 15 repetitions were performed, with each movement held for 10 s. The exercise intensity was increased after 4 weeks by adding weight with dumbbells.

**Figure 2:**
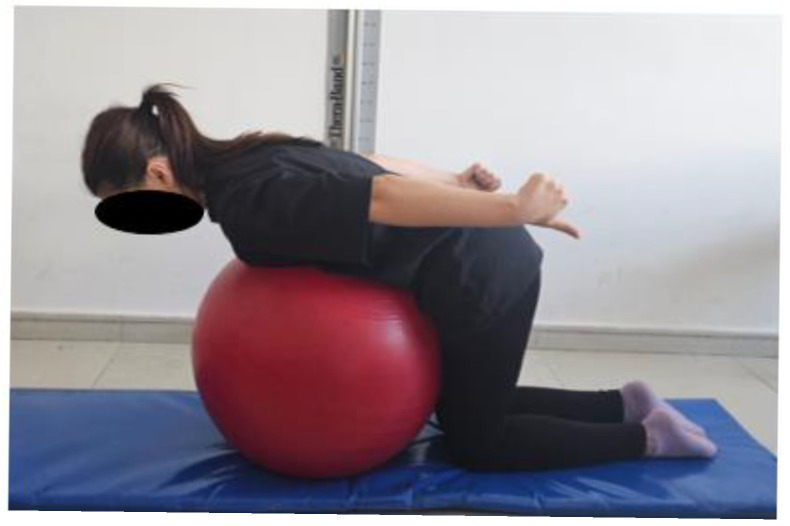
Scapular retraction exercise.

Scapular retraction exercise: The participants were asked to lift both arms backward while retracting both scapulae (Figure 2).

**Figure 3:**
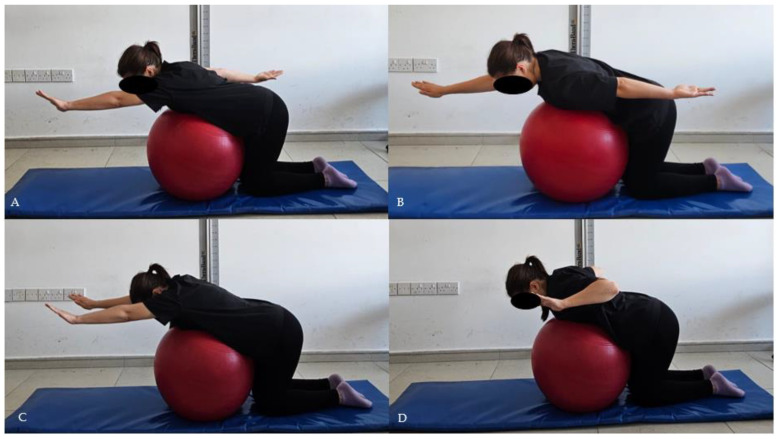
(**A**) Scapular dynamic stabilization exercises I starting position (**B**) Scapular dynamic stabilization exercises I finishing position (**C**) Scapular dynamic stabilization exercises II starting position (**D**) Scapular dynamic stabilization exercises II finishing position.

Scapular dynamic stabilization exercise I: The participants were asked to lift one arm to ear level and bring the other arm to pelvis level (Figure 3A). This movement progressed by adding weight. The same procedure was performed for the other arm (Figure 3B) [5].

Scapular dynamic stabilization exercise II: The participants were asked to lift both arms to head level (Figure 3C), then flex both elbows 90° to ear level (Figure 3D), and return to the starting position [5].

**Figure 4:**
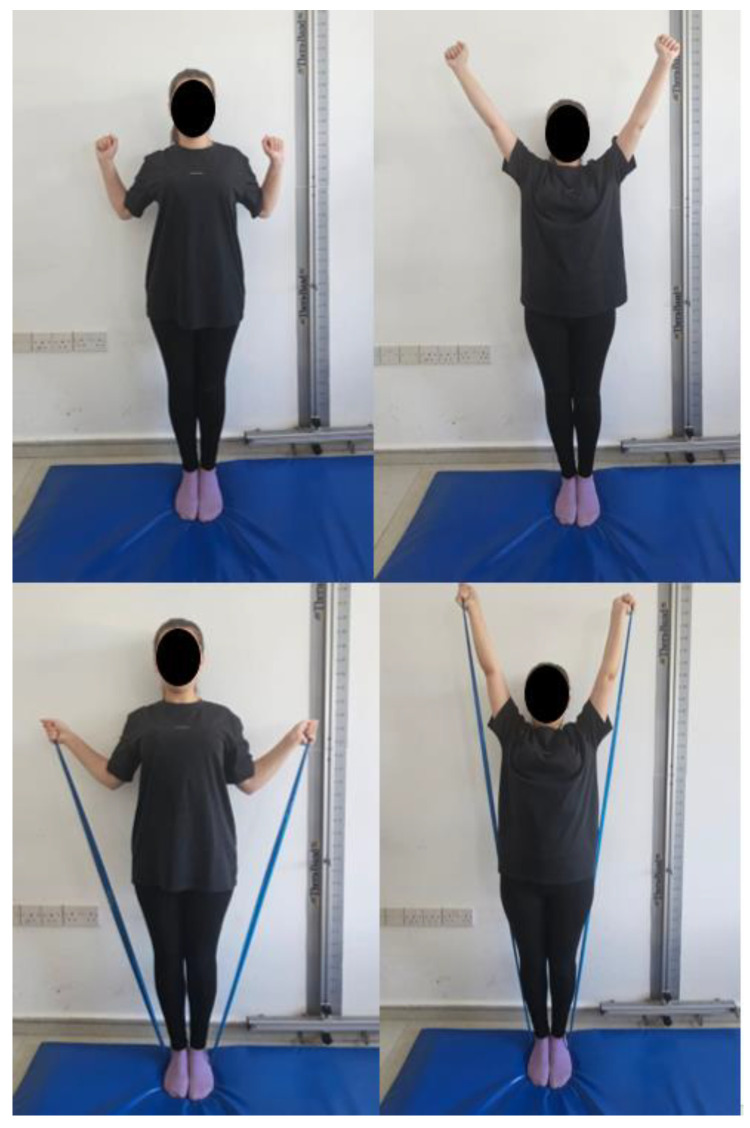
Scapular upward rotation exercises.

Scapular upward rotation exercises: The participants were asked to stand with their back against a wall, maintaining contact from the head to the buttocks, and feet positioned shoulder-width apart. In the starting position, the elbows were flexed at 90° and the shoulders were abducted to 90° with external rotation. The participants were instructed to slide their arms upward along the wall until reaching 180° of shoulder abduction. The participants were then instructed to maintain the arm position for 3 s. The exercise was conducted in three sets of 10–15 repetitions. These exercises were performed using an elastic exercise band (three sets of 10 repetitions) (Figure 4).

**Figure 5:**
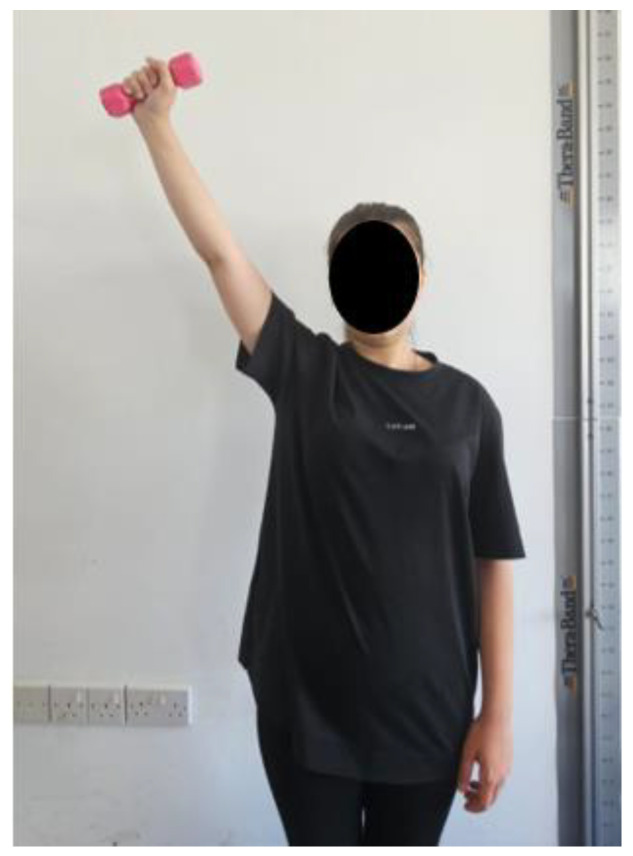
Arm raises for the lower trapezius muscle fibers.

Arm raises for the lower trapezius muscle fibers: The participants were asked to perform shoulder abduction in the scapular plane above 120° while standing (three sets of 10–15 repetitions). The exercise progressed by adding weight with dumbbells (three sets of 10 repetitions) (Figure 5). Moseley et al. identified shoulder abduction between 90° and 150° as the optimal exercise to activate the lower trapezius muscle (68% maximum voluntary isometric contraction). However, Ekstrom et al. discovered that shoulder abduction above 120° in the plane of the scapula led to 61% maximum voluntary isometric contraction EMG (electromyography) activity and was considered the best exercise. Shoulder abduction above 120° in the plane of the scapula is also an excellent exercise for coactivating the trapezius and serratus anterior muscles [20,21].

#### 2.4.3. Postural Stabilization Exercises

Lumbopelvic stabilization exercises began by teaching the participants a basic exercise using a stabilizer equipped with an air-inflated pressure sensor. The participants were placed supine, with knees flexed at 90°. A pressure cuff was placed under the lumbar spine and inflated to a pressure of 40 mm Hg. Exercises were initiated using the stabilizer to engage the primary muscles. We determined the level at which the pressure was kept constant while exercising (stable pelvis). The participants were first instructed in the “abdominal hollowing” exercise for co-contracting the transversus abdominis and multifidus muscles. The other exercises were continued to achieve motor control of the core muscles.

Bridge exercise: The participants were asked to perform a bridging exercise on the mat, with the head and shoulders supported on an exercise ball, pulling the cross elastic band with one hand across the trunk while remaining in this position. This exercise was practiced by lifting the legs one by one.

Abdominal sit-up exercise: The participants were asked to lie on their backs in the hook position. They were instructed to perform trunk flexion, with their hands clasped behind the neck, until the lower scapulae lifted off the floor.

Bird–dog exercise: First, the participants were asked to lift the right arm and left leg upward while keeping a crawling position. This process was repeated with the other leg and arm.

All lumbopelvic stabilization exercises were performed as 10–15 repetitions, with a 5 to 10 s contraction and a 5 s rest.

### 2.5. Outcome Measures

#### 2.5.1. Craniovertebral Angle Measurement

The craniovertebral angle (CVA) was formed via the intersection of a horizontal line passing through the seventh cervical vertebra in the sagittal plane and a line proceeding to the tragus of the ear [22,23]. The CVA was determined by marking certain anatomical points (ear tragus and C7 spinous process) of each person using anatomical markers. It was assessed in the standing position in the sagittal plane using photogrammetry [23,24,25,26]. Before taking the photographs, the neck was moved in the flexion–extension direction through the entire range of motion; this degree was gradually reduced, allowing the participants to regain their natural head–neck position [27]. The participants were then asked to stand upright with arms relaxed next to their trunks, feet placed 10 cm apart, and look straight ahead [28]. The markers were placed on the floor to maintain a consistent stance, ensuring the body was aligned 90° to the camera during each session. A landmark was placed on a white painted wall as the background [24,25]. A camera (Nikon D90, Nikon Corporation, Japan) was placed on a tripod 1.5 m away from the line indicating the position of the participant [27]. We adjusted the height of the tripod to ensure that the center of the objective lens was aligned with the acromion [29]. The photographs were transferred to a computer, where the marker points were connected using a paint program. The CVA was determined using a computer program (MB-Ruler v5.2—triangular screen ruler; Markus Bader-MB Softwaresolutions).

#### 2.5.2. Pain Assessment

The VAS was used to measure pain intensity. The participants were asked to mark their neck pain intensity experienced on a 0 to 10 cm horizontal line (0 indicating no pain and 10 representing the worst pain imaginable). The VAS displayed excellent test–retest reliability (ICC = 0.97) and strong validity (*r* = 0.71–0.78 with a 5-point verbal descriptive scale) for pain perception assessment. A change of at least two points was regarded as the minimum clinically important difference in individuals with chronic neck pain [30].

#### 2.5.3. Neck Disability Index

The NDI was administered to assess functional limitations due to chronic neck pain while performing daily activities. The questionnaire assessed the impact of cervical region-related pain on activities of daily life. It comprised ten questions, including four items on subjective symptoms, such as headache, pain intensity, concentration, and sleep, and six items on activities of daily living, such as personal care, weightlifting, work, reading, driving, and leisure activities. Each question scored 0–5, and individuals were asked to tick the most appropriate answer. The scores for all answers were added to obtain the total score. A low score indicated less impact, whereas a high score implied that the pain had more impact on the performance of daily activities [31]. The validity and reliability of the questionnaire were assessed by Vernom and Mior [31]. Turkish validity and reliability were assessed by Telci et al. in 2009 [32].

#### 2.5.4. Health-Related Quality of Life

The Turkish version of the 36-Item Short-Form Health Survey (SF-36) was used to evaluate health-related quality of life. SF-36 is a self-assessment scale that analyses eight dimensions of health, including physical functioning, social functioning, limitations for physical and emotional reasons, mental well-being, vitality (energy), pain, and general health perceptions, with 36 items. The worst and best scores are “0” and “100,” respectively. Higher scores imply better physical or mental health status. The Turkish validity and reliability of the questionnaire were assessed by Kocyigit et al. [33].

### 2.6. Statistical Analysis

Descriptive statistics were calculated, and the Shapiro–Wilk test was used to check the normality. Continuous variables were expressed as mean t standard de E (Ct) − x + SD) and median (IQR interquartile range). The independent samples t test was used to compare the demographic and physical characteristics of the participants. To assess changes over time and between groups, a two-way repeated measures analysis of variance (ANOVA) was conducted, with group (SLPS vs. CS) as the between-subjects factor and time (pre-treatment, post-treatment, follow-up) as the within-subjects factor. When significant interaction effects were detected, Sidák’s multiple comparisons test was applied as a post hoc procedure to identify specific differences within and between groups across time points. A *p*-value of less than 0.05 was considered statistically significant. Data were analyzed using SPSS 22.0 software (SPSS Inc., Chicago, IL, USA) and Graphpad Prism software (Demo version 9.5.0 (525), Graphpad Software, San Diego, CA, USA) for macOs. For between-group comparisons, effect sizes were calculated using Cohen’s d, along with 95% confidence intervals (CIs) for mean differences. Additionally, partial eta-squared (η^2^) values were reported to quantify the magnitude of group and interaction effects derived from the two-way repeated measures ANOVA.

## 3. Results

The socio-demographic and clinical baseline characteristics of the individuals in the SLPS and CS groups are shown in Table 1, and the groups were found to be similar (*p* > 0.05).

Table 2 shows the VAS, CVA, and NDI comparison of the study groups at three different measurement points. At baseline, there were no statistically significant differences between the SLPS and CS groups in terms of pain intensity (VAS), CVA, or NDI (*p* > 0.05 for all comparisons). Post-treatment measurements revealed significant differences favoring the SLPS group. The VAS scores were markedly lower in the SLPS group than in the CS group both immediately after treatment (0.93 ± 1.36 vs. 4.60 ± 1.79; mean difference = −3.67, 95% CI [−4.49, −2.85], *p* < 0.001; Cohen’s d = −2.31; η^2^ = 0.48) and at follow-up (2.60 ± 1.54 vs. 6.27 ± 1.98; mean difference = −3.67, 95% CI [−4.59, −2.75]; *p* < 0.001; Cohen’s d = −2.07; η^2^ = 0.43). The CVA was significantly greater in the SLPS group at both the post-treatment (58.00 ± 4.41 vs. 53.00 ± 4.48; mean difference = 5.00, 95% CI [2.70, 7.30]; *p* < 0.001; Cohen’s d = 1.12; η^2^ = 0.17) and follow-up (56.50 ± 4.66 vs. 51.59 ± 4.36; mean difference = 4.91, 95% CI [2.58, 7.24]; *p* < 0.001; Cohen’s d = 1.09; η^2^ = 0.16) assessments. Similarly, the NDI was significantly lower in the SLPS group post-treatment (3.73 ± 2.21 vs. 12.43 ± 7.61; mean difference = −8.70, 95% CI [−11.60, −5.80]; *p* < 0.001; Cohen’s d = −1.55; η^2^ = 0.29) and at follow-up (6.73 ± 2.83 vs. 15.07 ± 8.23; mean difference = −8.34, 95% CI [−11.52, −5.16]; *p* < 0.001; Cohen’s d = −1.36; η^2^ = 0.25), indicating less perceived disability.

In Table 3, the SF-36 scale and subscale score comparisons of the study groups at three different measurement points are shown. At baseline measurement, there were no statistically significant differences for any of the SF-36 subscales (*p* > 0.05 for all comparisons).

However, in the physical functioning subscale, the SLPS group reported higher scores than the CS group post-treatment (86.67 ± 10.69 vs. 75.17 ± 21.27; mean difference = 11.50, 95% CI [2.80, 20.20]; *p* = 0.034; Cohen’s d = 0.68; η^2^ = 0.10) and at follow-up (84.83 ± 12.07 vs. 72.83 ± 23.37; mean difference = 12.00, 95% CI [2.39, 21.61]; *p* = 0.048; Cohen’s d = 0.65; η^2^ = 0.09). For role limitations due to physical health, the SLPS group scored significantly higher both post-treatment (89.17 ± 16.97 vs. 60.00 ± 32.56; mean difference = 29.17, 95% CI [15.75, 42.59]; *p* < 0.001; Cohen’s d = 1.12; η^2^ = 0.26) and at follow-up (75.83 ± 22.25 vs. 50.83 ± 31.82; mean difference = 25.00, 95% CI [10.81, 39.19]; *p* = 0.003; Cohen’s d = 0.91; η^2^ = 0.19). A similar pattern was observed in the subscale of role limitations due to emotional problems, with the SLPS group scoring higher than the CS group post-treatment (88.23 ± 18.16 vs. 54.45 ± 37.64; mean difference = 33.78, 95% CI [18.51, 49.05]; *p* < 0.001; Cohen’s d = 1.14; η^2^ = 0.27) and at follow-up (71.14 ± 22.69 vs. 39.60 ± 41.82; mean difference = 31.54, 95% CI [14.15, 48.93]; *p* = 0.002; Cohen’s d = 0.94; η^2^ = 0.20).

In the energy/fatigue subscale, the SLPS group demonstrated significantly better scores at both time points after the intervention (post-treatment: 67.00 ± 15.35 vs. 51.00 ± 20.53, mean difference = 16.00, 95% CI [6.63, 25.37], *p* = 0.004; Cohen’s d = 0.88; η^2^ = 0.17; follow-up: 64.67 ± 14.26 vs. 47.50 ± 21.08, mean difference = 17.17, 95% CI [7.87, 26.47], *p* = 0.002; Cohen’s d = 0.95; η^2^ = 0.20). Emotional well-being was also significantly higher in the SLPS group post-treatment (71.53 ± 16.01 vs. 58.33 ± 21.48; mean difference = 13.20, 95% CI [3.41, 22.99]; *p* = 0.028; Cohen’s d = 0.70; η^2^ = 0.12), although the difference was not significant at follow-up (*p* = 0.127). Regarding social functioning, post-treatment scores favored the SLPS group (90.87 ± 16.69 vs. 70.98 ± 27.50; mean difference = 19.89, 95% CI [8.14, 31.64]; *p* = 0.004; Cohen’s d = 0.87; η^2^ = 0.17), and this difference remained significant at follow-up (82.27 ± 15.77 vs. 62.25 ± 29.94; mean difference = 20.02, 95% CI [7.66, 32.38]; *p* = 0.007; Cohen’s d = 0.84; η^2^ = 0.15). In the pain subscale, scores were significantly higher in the SLPS group than in the CS group both post-treatment (87.58 ± 9.88 vs. 61.23 ± 19.45; mean difference = 26.35, 95% CI [18.38, 34.32]; *p* < 0.001; Cohen’s d = 1.71; η^2^ = 0.47) and at follow-up (75.42 ± 10.71 vs. 54.17 ± 19.52; mean difference = 21.25, 95% CI [13.11, 29.39]; *p* < 0.001; Cohen’s d = 1.35; η^2^ = 0.38). General health scores did not differ significantly between groups at any time point (*p* > 0.05).

## 4. Discussion

This study investigated the effects of SLPS exercises in addition to CS on the CVA, disability, and quality of life in people with chronic neck pain. The results showed that including SLPS exercises in the treatment program, rather than limiting the treatment to cervical exercises alone, led to significant improvements in cervical posture, neck disability, pain, and health-related quality of life. The demographic characteristics of the participants in the groups (age, height, weight, and body mass index) were recorded. The demographic characteristics showed no significant difference between the groups. Previous studies have demonstrated a higher prevalence of neck pain in people aged more than 40 years. The mean age of the participants in this study was 37.5 and 38.1 years in the SLPS and CS groups, respectively. The findings of this study are consistent with the published results in terms of age range [34,35].

Suvarnnato et al. [36] compared the effects of training the semispinalis cervicis and deep cervical flexor muscles and usual care (control) on the CVA in people with chronic neck pain. Participants with chronic neck pain in this study were categorized into three groups. The participants in the first, second, and third groups were treated with semispinalis cervicis isometric exercises, deep cervical flexion exercises with a pressure stabilizer, and a conservative physiotherapy approach, respectively. The CVA was evaluated by photography, similar to our study. At the end of the study, exercises for cervical flexor and extensor muscle groups were found to be more effective at increasing the CVA [36]. This result is similar to our results. In our study, both the SLPS and CS exercises were found to increase CVA, but CVA increased more in the SLPS group than in the CS group.

In a study conducted by Kang and Kyoung [37], teachers with chronic neck pain with a CVA of less than 53° were treated with scapular stabilization and thoracic extension exercises in one group, and the other group received cervical myofascial release and stretching exercises. The participants performed these exercises three times a week for 6 weeks. The results showed a significant increase in the CVA in the group performing scapular stabilization and thoracic extension exercises compared with the other group. Exercises targeting the scapula and thoracic spine were more effective than those targeting the neck structure directly [37]. Another study, conducted by Kang et al. [38], reported that scapular stabilization exercises increased the CVA by activating the neck muscles, lower trapezius, and serratus anterior muscle. In parallel with this study, our study demonstrated a significant increase in the CVA values in the SLPS group, including cervical, scapular, and lumbopelvic stabilization exercises, compared with the exercise group that included only neck exercises. When we look at the literature, it shows us that in individuals with neck pain, it is not only the cervical region that should be treated—the columna vertebralis should be considered as a whole [38]. Therefore, including SLPS training in the treatment of chronic neck pain will be beneficial in increasing the CVA.

Kang and Beomryong compared cervical and scapular-focused resistance exercises with trapezius massage in patients with chronic neck pain [39]. The people participating in the study were divided into two groups. The first group was treated with resistance exercises focused on cervical and scapular muscles, whereas the second group was treated with trapezius massage. Exercises were performed five times a week for 4 weeks in both groups. The NDI was used to assess the level of disability, and SF-36 was used to assess the quality of life among the study participants. Participants who performed cervical and scapular resistance exercises showed greater improvement in disability levels than participants who did not receive trapezius massage. Participants who performed cervical and scapular resistance exercises showed an increase in SF-36 physical component and SF-36 mental component scores [39]. In our study, one group was given resistance exercises for the cervical and scapular region, as in the study by Kang and Beomryong. However, we added lumbopelvic exercises to these exercises. Therefore, in our study, the disability score was lower and SF-36 parameters were higher in the SLPS group after treatment and in the follow-up measurements.

The onset or persistence of chronic neck pain may be attributed to instability of the muscles around the scapulothoracic region. It may also be due to impaired control of the muscles surrounding the scapulothoracic region [40]. The muscles around the scapulothoracic region play a crucial role in clinically managing people with neck pain. Clinically, the shortening of the upper trapezius muscle and the weakening of the lower trapezius muscle lead to muscle imbalance around the scapulothoracic region. The strength and endurance of the lower trapezius muscle are reduced in individuals with neck pain [40,41]. Strengthening exercises targeting the scapulothoracic muscles increase muscle strength, decrease muscle imbalance, and improve scapulothoracic posture [42]. However, studies on interventions targeting imbalance in the muscles of this region aiming to strengthen weakened scapulothoracic muscles in people with neck pain are scarce. Spinal pain impacts core muscle control in people with neck pain, and core muscle function associated with neck pain may change [11]. People with neck pain have abnormal performance in lumbar stabilization [12]. In our study, the SLPS group demonstrated a greater reduction in pain and disability levels with applications to the muscles in the scapular and lumbopelvic regions than the CS group.

In the study conducted by Ganu and Gor, individuals with neck pain were categorized into two groups. Individuals in the first group underwent scapular stabilization exercises without abdominal control feedback for 6 weeks. Individuals in the second group underwent scapular stabilization exercises with abdominal control feedback for 6 weeks. The primary objective of this study was to assess the effects of including abdominal control feedback in the traditional scapular stabilization exercise protocol. Abdominal control feedback involves voluntary abdominal pulling by exercising the transversus abdominis, a deep abdominal muscle. The study reported a significant improvement in pain on adding the abdominal control feedback protocol to the traditional protocol [43]. In our study, SLPS training was found to be more effective in reducing pain and disability than CS. In the 2-month follow-up evaluation, it was found that pain was reduced in the SLPS group compared to the CS group. SLPS training was more effective at improving pain in people with chronic neck pain.

The mechanism underlying the positive impact of exercise on pain is that exercise reduces the cycle of muscle tension, metabolite buildup and impaired circulation, and myofascial pain by resetting the intrafusal fibers. Exercise programs reduce pain by enhancing intermuscular function. Functional balance between muscles is crucial. Motor control training enhances coordination between deep cervical flexors, superficial cervical flexors, and deep lumbar spine muscles. Moreover, strong isometric neck muscle contractions stimulate muscle stretch receptors and trigger the release of beta-endorphins from the pituitary gland, resulting in pain reduction [44,45]. This explains why pain decreased more in the SLPS group than in the CS group and emphasizes the importance of exercise in the rehabilitation program.

For neck pain, a minimum clinically significant difference of 10 points on the SF-36 quality-of-life scale is considered meaningful. In our study, the measurements made after providing treatment in the SLPS group were consistent with the available findings [46].

Our study shows that adding SLPS exercises to the rehabilitation program is clinically useful for reducing pain and disability and increasing quality of life and the CVA in individuals with chronic neck pain. It also shows that in individuals with neck pain, not only the muscles in the cervical region but also the columna vertebralis as a whole should be considered, and the muscles in the scapular and lumbopelvic regions should also be treated. This will shed light on future studies.

## 5. Limitations

This study has some limitations. First, the assessments were performed at different times of day, and no standardization was determined for the evaluation time. Second, the same principle investigator was responsible for the randomization, administration of the assessment, and treatment. Third, this study was unblinded, as both the participants and the physiotherapist needed to know the applied exercise protocol. These factors may have introduced potential performance and observer bias. However, efforts were made to minimize these risks.

## 6. Conclusions

The inclusion of SLPS training in the treatment program for patients with chronic neck pain led to a significant improvement in forward head posture. Moreover, comprehensive postural stabilization training resulted in much more improvement in neck disability and the quality of life of people with neck pain than the training consisting only of CS. Combining SLPS training with neck stabilization exercises can positively influence the clinical outcomes of patients with neck pain.

## Figures and Tables

**Table 1:** Demographic and physical characteristics of patients with chronic neck pain participating in the study.

Variable	SLPS Group (*n* = 30) X ± SD	CS Group (*n* = 30) X ± SD	*p*-Value
Age (year)	37.50 ± 13.14	38.16 ± 14.33	0.852
Height (cm)	163.6 ± 5.88	163.0 ± 5.75	0.691
Weight (kg)	67.16 ± 12.10	65.40 ± 13.77	0.600
BMI (kg/m^2^)	24.80 ± 3.70	24.70 ± 4.34	0.919
Working time (year)	12.06 ± 11.46	13.60 ± 12.09	0.616

Independent-sample *t* test; *p*-value significant at or less than 0.05. BMI, body mass index; CS, cervical stabilization; SLPS, scapular and lumbopelvic stabilization.

**Table 2:** VAS, CVA, and NDI comparison of the study groups at three different measurement points.

		SLPS Group (*n* = 30)		CS Group (*n* = 30)		p^2^
		$\bar{x}$ ± SD	Median (IQR)	$\bar{x}$ ± SD	Median (IQR)	
VAS	Pre-treatment	6.43 ± 1.81	6.50 (3.00)	6.13 ± 2.00	5.00 (3.00)	0.906
	Post-treatment	0.93 ± 1.36	0.00 (2.00)	4.60 ± 1.79	5.00 (3.00)	<0.001
	Follow-Up	2.60 ± 1.54	3.00 (3.00)	6.27 ± 1.98	7.00 (3.00)	<0.001
p^1^		a, b, c		a, c		
CVA (°)	Pre-treatment	50.35 ± 4.76	50.33 (7.17)	51.24 ± 4.47	51.89 (6.76)	0.838
	Post-treatment	58.00 ± 4.41	56.91 (7.54)	53.00 ± 4.48	53.47 (5.66)	<0.001
	Follow-Up	56.50 ± 4.66	56.25 (7.65)	51.59 ± 4.36	52.56 (4.97)	<0.001
p^1^		a, b, c		a, c		
NDI	Pre-treatment	18.00 ± 7.25	19.00 (9.00)	16.17 ± 8.54	14.50 (11.00)	0.755
	Post-treatment	3.73 ± 2.21	3.00 (3.00)	12.43 ± 7.61	11.00 (11.00)	<0.001
	Follow-Up	6.73 ± 2.83	7.00 (4.00)	15.07 ± 8.23	13.00 (11.00)	<0.001
p^1^		a, b, c		a, c		

p^1^: within-group comparisons; p^2^: between-group comparison; a: statistically significant difference between pre- and post-treatment; b: statistically significant difference between pre-treatment and follow-up; c: statistically significant difference between post-treatment and follow-up. VAS: Visual Analog Scale, NDI: Neck Disability Index.

**Table 3:** SF-36 scale and subscale comparison of the study groups at three different measurement points.

		SLPS Group (*n* = 30)		CS Group (*n* = 30)		p^2^
		$\bar{x}$ ± SD	Median (IQR)	$\bar{x}$ ± SD	Median (IQR)	
SF-36 Physical functioning	Pre-treatment	72.33 ± 16.39	72.50 (20.00)	69.17 ± 27.76	77.50 (45.00)	0.933
	Post-treatment	86.67 ± 10.69	90.00 (15.00)	75.17 ± 21.27	80.00 (30.00)	0.034
	Follow-Up	84.83 ± 12.07	87.50 (15.00)	72.83 ± 23.37	80.00 (30.00)	0.048
p^1^		a, b, c		a		
SF-36 Role limitations due to physical health	Pre-treatment	52.50 ± 39.03	50.00 (75.00)	49.17 ± 40.73	50.00 (100.00)	0.984
	Post-treatment	89.17 ± 16.97	100.00 (25.00)	60.00 ± 32.56	50.00 (50.00)	<0.001
	Follow-Up	75.83 ± 22.25	75.00 (50.00)	50.83 ± 31.82	50.00 (50.00)	0.003
p^1^		a, b, c		c		
SF-36 Role limitations due to emotional problems	Pre-treatment	51.12 ± 43.54	50.10 (100.00)	47.78 ± 43.49	33.30 (100.00)	0.987
	Post-treatment	88.23 ± 18.16	100.00 (33.30)	54.45 ± 37.64	66.70 (66.70)	<0.001
	Follow-Up	71.14 ± 22.69	66.70 (33.30)	39.60 ± 41.82	33.30 (66.70)	0.002
p^1^		a, b, c		c		
SF-36 Energy/fatigue	Pre-treatment	40.00 ± 21.24	40.00 (25.00)	43.67 ± 24.81	42.50 (40.00)	0.903
	Post-treatment	67.00 ± 15.35	65.00 (15.00)	51.00 ± 20.53	50.00 (20.00)	0.004
	Follow-Up	64.67 ± 14.26	62.50 (15.00)	47.50 ± 21.08	50.00 (30.00)	0.002
p^1^		a, b		a		
SF-36 Emotional well-being	Pre-treatment	56.53 ± 16.16	54.00 (20.00)	56.53 ± 24.13	60.00 (40.00)	>0.999
	Post-treatment	71.53 ± 16.01	72.00 (12.00)	58.33 ± 21.48	58.00 (36.00)	0.028
	Follow-Up	66.60 ± 15.14	68.00 (12.00)	56.67 ± 21.64	60.00 (24.00)	0.127
p^1^		a, b, c		-		
SF-36 Social functioning	Pre-treatment	72.25 ± 20.09	75.00 (37.50)	66.67 ± 30.68	75.00 (37.50)	0.793
	Post-treatment	90.87 ± 16.69	100.00 (12.50)	70.98 ± 27.50	75.00 (50.00)	0.004
	Follow-Up	82.27 ± 15.77	87.50 (12.50)	62.25 ± 29.94	62.50 (37.50)	0.007
p^1^		a, c		c		
SF-36 Pain	Pre-treatment	51.58 ± 19.49	46.25 (25.00)	56.67 ± 23.06	57.50 (32.50)	0.738
	Post-treatment	87.58 ± 9.88	90.00 (20.00)	61.23 ± 19.45	67.50 (30.00)	<0.001
	Follow-Up	75.42 ± 10.71	77.50 (12.50)	54.17 ± 19.52	55.00 (22.50)	<0.001
p^1^		a, b, c		c		
SF-36 General health	Pre-treatment	50.17 ± 18.55	47.50 (20.00)	57.17 ± 19.81	55.00 (30.00)	0.414
	Post-treatment	66.17 ± 19.15	62.50 (25.00)	58.00 ± 22.38	55.00 (35.00)	0.352
	Follow-Up	61.83 ± 16.99	60.00 (25.00)	55.33 ± 20.04	50.00 (30.00)	0.450
p^1^		a, b, c		-		

p^1^: within-group comparisons; p^2^: between-group comparison; a: statistically significant difference between pre- and post-treatment; b: statistically significant difference between pre-treatment and follow-up; c: statistically significant difference between post-treatment and follow-up. SF-36: Short-Form Health Survey.

## Data Availability

Data are contained within this article. The data presented in this study are available upon request from the corresponding author due to ethical reasons.

## Abbreviations

The following abbreviations are used in this manuscript:

CS	Cervical stabilization group
CVA	Craniovertebral angle
EMG	Electromyography
NDI	Neck disability index
SF-36	Short-Form Health Survey
SLPS	Scapular and lumbopelvic stabilization
VAS	Visual analog scale
